# Dyes Used in Processed Meat Products in the Polish Market, and Their Possible Risks and Benefits for Consumer Health

**DOI:** 10.3390/foods12132610

**Published:** 2023-07-06

**Authors:** Katarzyna Czech-Załubska, Daniel Klich, Agnieszka Jackowska-Tracz, Anna Didkowska, Janusz Bogdan, Krzysztof Anusz

**Affiliations:** 1 Department of Food Hygiene and Public Health Protection, Institute of Veterinary Medicine, Warsaw University of Life Sciences (SGGW), Nowoursynowska 166, 02-787 Warsaw, Poland; katarzyna_czech_zalubska@sggw.edu.pl (K.C.-Z.); agnieszka_jackowska_tracz@sggw.edu.pl (A.J.-T.); janusz_bogdan@sggw.edu.pl (J.B.); krzysztof_anusz@sggw.edu.pl (K.A.); 2 Institute of Animal Sciences, University of Life Sciences—SGGW, Ciszewskiego 8, 02-786 Warsaw, Poland; daniel_klich@sggw.edu.pl

**Keywords:** annatto, betanin, caramel, carmine, dyes, food additives, food safety, paprika extract

## Abstract

Manufacturers are obliged to label processed meat products with information concerning the additives used and nutritional values. The aim of the study was to identify the dyes most frequently used in processed meat, evaluate their influence on specific food qualities, assess whether their use was correct and review their effect on health. The analysis was based on information on the labels and images of processed meat, and used a generalised linear model with a binary dependent variable. The risks and benefits for human health were defined based on the available literature. Twelve dyes were found to be used in the manufacture of processed meat. Carmine was found in 183 of 273 (67.03%) evaluated assortments containing dyes. The occurrence of water, flavourings and high fat and carbohydrate contents increased the chances that a dye would be present in a particular product. Unauthorised use of food additives was found in 20 products, with smoked meat products demonstrating the highest number of non-compliances. In general, the dyes used with food are considered safe; however, reservations are associated with the use of E150C and E150D caramels due to their potential carcinogenic effect, and carmine and annatto due to their allergic effects.

## 1. Introduction

The decision to purchase a particular food item is strongly influenced by its appearance [1,2,3]. One such quality is food colour, which may be interpreted as an indicator of flavour, freshness, maturity or wholesomeness, and its intensity may also affect taste perception [2,4,5]. Therefore, food manufacturers often employ additives to improve the colour of their products and make them more attractive to consumers.

Even though colour may be one of the most important considerations in a purchasing decision, it should be emphasised that food must primarily be safe for consumption [6]. Any unauthorised use of food additives may seriously affect human health.

The use of pigments and other food additives by food manufacturers within the borders of the European Union is regulated by Regulation 1333/2008 of the European Parliament and Council (EC) of the 16 December 2008 regarding food additives [7]. The legislator has authorised 41 additives, classified as pigments, based on their role in the final product. In addition, to more precisely define the conditions permitting the use of additives, foods have been divided into specific categories [8]. The present study concerns products within the following categories as defined in Regulation No. 853/2004 (EC): Meat preparations (No. 8.2) and Meat products (No. 8.3). The latter is divided into Non-heat-treated processed meat (No. 8.3.1) and “Heat-treated processed meat” (No. 8.3.2) [7,8,9]. Depending on the food category and substance type, pigment use can be determined by the level of *quantum satis* or the maximum numerical value set by the legislator [7].

Legislation on the use of additives varies between different parts of the world. In the US, general rules for using food colours are regulated by § 70 Title 21 of the Code of Federal Regulations [10]. As a result, nine food additives acting as pigments have jointly been certified and approved for use in the food industry by the Food and Drug Administration (FDA), seven of which are intended for general use [11].

The aim of the present study is to identify the most common dyes present in processed meat on the Polish market, examine the relationships between their presence and the food characteristics, and evaluate their correctness of use; these aims are achieved by an analysis of information of product labels. Based on the available literature, it also assesses the risks and benefits to human health of using such dyes. Knowledge of the presence of dyes in meat products and meat preparations may also affect the dietary and purchasing decisions among consumers predisposed to allergic reactions.

## 2. Materials and Methods

### 2.1. Study Design

The assessment included only processed meat products. Based on the label description and an image of the product, the product was categorised as a meat preparation (8.2) or meat product (8.3) as defined in Regulation 853/2004 (EC) [9]. Next, due to their considerable variety, the meat products were then subdivided into four groups (smoked meats, sausages, offal meat and other meat products) according to Polish Standard PN-A-82007, “Przetwory mięsne. Wędliny.” (*Meat products. Smoked*) [12]. Therefore, the analysis was conducted in a total of five groups.

The analysis encompassed labels from meat products and meat preparations found on shelves and in display fridges at shops included in the study. All meat preparations and most of the meat products (all smoked meat, most of the sausages, pates and other meat products, excluding those listed below) were chilled and stored at a temperature of 2–10 degrees. Some kabanos sausages, pate and canned ham were stored at no higher than 25 degrees ambient temperature. These were obtained from the five largest retail chains offering the above ranges of goods, based on their share in the market according to total income in 2018, as indicated by the Ministry of Finance report for 2019 [13]. Those were, in decreasing order of income, Biedronka (JERONIMO MARTINS POLSKA), Lidl (FRF Beteiligungs GmbH), Eurocash (a conglomerate comprising Lewiatan, Groszek and Delikatesy Centrum), as well as the Auchan and Kaufland networks.

The study sites comprised all Polish cities with populations exceeding 250,000 inhabitants (11 cities) [14] as well as six minor cities. In each of the 17 cities, one representative per retail network mentioned above was selected, provided that the shops of that particular network were present and the samples were collected there. Photographs of the product labels were taken for analysis, together with the products themselves.

In total, 12,333 labels were analysed, all of which were available in the shops during the study. The labels were collected over a few months, from October 2020 to March 2021, in 75 shops in 17 Polish cities.

### 2.2. Statistical Analysis

A generalised linear model with a binary dependent variable was constructed to predict the occurrence of dyes in processed meat products based on [15].

Two such analyses were carried out, the first including all processed meat products analysed in the study and the second including only sausages, as this group presented the highest frequency of dyes. In the all-products model, the dependent variable was the presence of a dye in the product, marked as 1, and the lack of a dye, marked as 0. The explanatory variables were product features that were obtained from the information on the labels, including (1) food technology groups (divided into five main groups: meat preparations, smoked meats, sausages, offal meat and other meat products); (2) water as an ingredient (two categories: lack and presence of water); (3) flavours (two categories: lack and presence of flavours); and (4) covariates: protein (g/100 g of product), carbohydrates (g/100 g of product) and fat (g/100 g of product).

Covariates were first assessed for collinearity using Pearson’s pairwise correlation. The meat content in the product (g/100 g of the product) was not included, due to its high correlation with protein content, nor was the type of meat (divided into poultry, pork and beef), due to quasi-complete separation.

The second model, sausages, used the same dependent variable and independent variables; however, the product variable was omitted. Both models were compared with the null *intercept-only* model to verify their explanatory power.

Calculations were based on data concerning the nutritional values of respective lots; as such, products lacking complete data were excluded from the analysis regarding the prediction of dye presence in processed meat based on product qualities. Therefore, 994 records were included in the study.

The risks to consumer health, and the potential benefits, were assessed based on the literature within the National Library of Medicine and the National Centre for Biotechnology Information, i.e., pubmed.gov databases. The database was searched for names of additives. Preliminary verification of publications was carried out based on their abstracts. A total of 134 papers were qualified for further analysis. After reviewing the entire publication, 43 of these papers were excluded due to missing desired information. Following this, the content of the 91 remaining articles was then evaluated. A ranking system was created to signify each publication in terms of the occurrence of risks and benefits for human health:

- Score = 0 (no risk or benefits): the papers indicate that the dye has no genotoxicity or carcinogenicity, acute or chronic toxicity or hypersensitisation potential but also has no benefit to human health
- Score = 1 (occurrences of risk or benefits): the papers indicate genotoxicity, carcinogenicity, acute or chronic toxicity, or hypersensitisation potential or benefit for human health concerning a particular dye.

Therefore, each dye was assigned a specific number of points for each of the five risks or benefits. Then, the ratio of the number of publications indicating the occurrence of a given risk/benefit to a specific dye to all publications about a given risk/benefit concerning a particular dye was calculated.

## 3. Results

### 3.1. Incidence

In total, 1967 unique assortments belonging to food categories 8.2, 8.3.1 and 8.3.2 were identified. Of these, 273 (13.88%) contained additives that manufacturers indicated were dyes. Moreover, more than one pigment was used for the production of 31 (1.6%) assortments, and at least three dyes were used in the case of four (0.2%) assortments.

**Figure 1:**
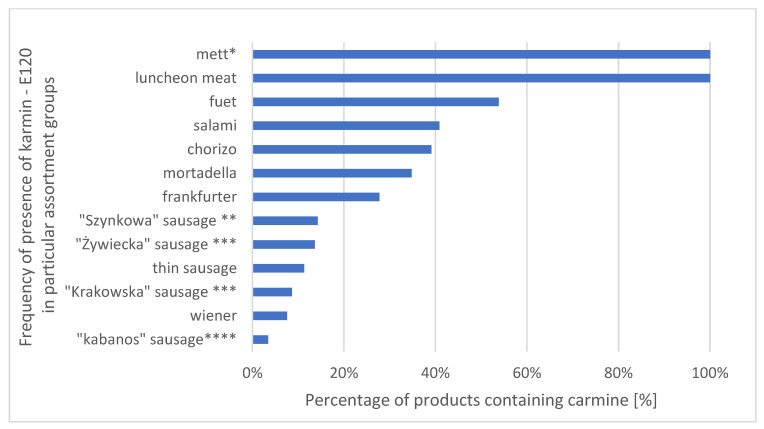
Frequency of presence of carmine (E120) in processed meat belonging to particular food products. Product descriptions: * a preparation of seasoned minced raw pork that is popular in Germany and Poland; ** a sausage prepared using ham and other ingredients, the latter varying by location. It is part of the cuisines of China, Germany, Poland and the United States; *** a type of Polish sausage, usually served as a cold cut; **** a long, thin, dry sausage traditionally made of pork that originated in Poland.

A total of 12 food additives acting as dyes were identified in the studied meat products and preparations available in the Polish market. The most frequently used dye was carminic acid (E120), which was found in 183 out of the 273 assortments containing dyes (67.03%) (Figure 1). Carbon (E153), carotenes (E160A) and titanium dioxide (E171) (Table 1) were the rarest dyes, being identified in just single lots.

### 3.2. The Analysis of Label Incompliance

In the case of food dyes, it is impossible to unambiguously determine the correctness of their use in processed meat only based on labels. Some substances are not authorised for use as dyes in foods belonging to food categories 8.2 (Meat preparations), 8.3.1 (Non-heat-treated processed meat) and 8.3.2 (Heat-treated processed meat), with a concomitant admission for use in category 8.3.3 (Casings, coatings and decorations for meat). Therefore, an additional visual assessment was performed of products that did not meet the requirements of use designed for food categories 8.2, 8.3.1 and 8.3.2. If the dye was found to be part of an edible casing or decoration on the surface of the analysed product, its use was classed as compliant with regulations. The remaining products without decoration nor edible casing were considered potentially non-compliant. These findings are presented in Table 2. No such improper use was observed for curcumin (E100), carotenes (E160A) and annatto (E160B).

Potential non-compliance for riboflavin (E101) and paprika extract (E160C) was observed in one out of four and one out of thirty-one products, respectively. In addition, potential non-conformance was noted in 7 out of 183 products containing carminic acid, 8 out of 38 examples of E150A-E150D caramels and in 1 out of 29 cases of betanin.

Possible non-compliance was noted in the case of E153 (carbon). E153 is not authorised for use in foods in categories 8.2, 8.3.1 and 8.3.2; however, it is authorised for use in category 8.3.3. As visual assessment showed that the product tested had neither edible casing nor meat decoration, the product was classified as non-compliant.

Products containing colour E171 were classified as non-compliant, as this colour is no longer authorised for use in food.

Out of all analysed assortment groups, the highest number of non-compliances was noted in smoked cold meats. The additives with the highest percentage of non-compliances were riboflavin and the caramel group.

### 3.3. An Analysis of Dye Presence Prediction in Processed Meat Based on Product Qualities

**Figure 2:**
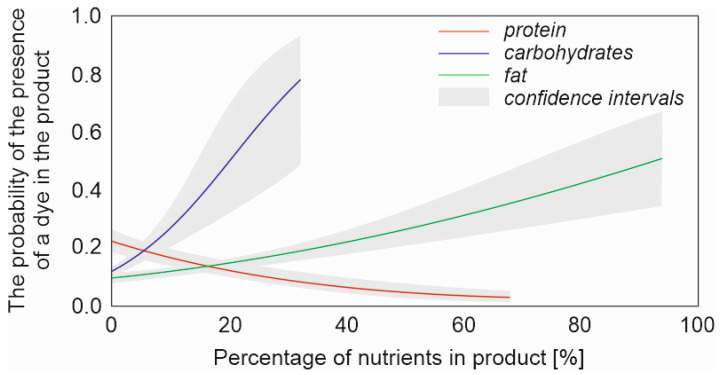
The probability of the presence of a dye in meat product against the percentage of nutrients (protein, carbohydrates and fat).

The presence of dyes in processed meat was significantly dependent on the type of product (PRODUCT) (Table 3). Sausages were over six times more likely to include dyes compared to smoked meats (B = 1.901, *p* < 0.001), while other meat products were almost three times more likely (B = 1.068, *p* = 0.005). The presence of dyes could also be predicted by the presence of water (*p* < 0.001) and flavours (*p* = 0.001) in the products; however, in both cases, the lack of this component (water and flavours) resulted in a slightly lower chance of a dye being present (B = −0.591 and B = −0.494, respectively). The content of nutrients in the product could also predict the presence of dyes. The chance of a dye being present increased with the increase in fat and carbohydrate content per 100 g of the product (B = 0.031, *p* < 0.00 and B = 0.092, *p* = 0.001, respectively). However, the likelihood of a dye decreased with the increase in the protein content (B = 0.070, *p* < 0.001). Carbohydrate content had the greatest effect of the three covariates studied (Figure 2).

**Figure 3:**
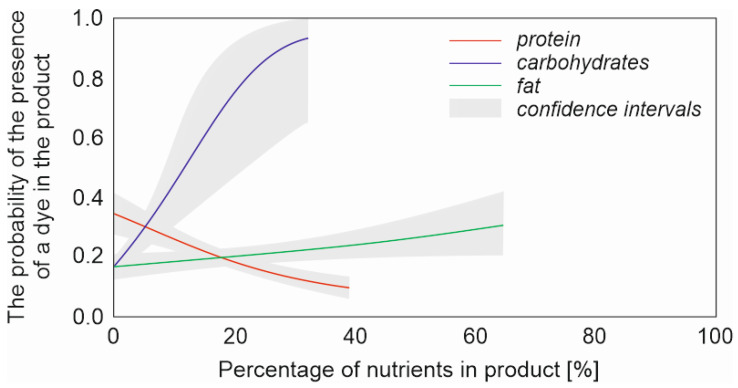
Probability of presence of dye in sausages against the percentage of nutrients (protein, carbohydrates and fat).

Similar trends regarding the occurrence of dyes were observed in the analysis for sausages (Table 3). Similarly, a lack of water and flavours indicated a lower chance of dyes (B = −0.561, *p* = 0.004 and B = −0.466, *p* = 0.011, respectively). There was also a similar relationship with nutrients, i.e., the chance of dyes increased with fat and carbohydrate content (B = 0.058, *p* < 0.001 and B = 0.093, *p* = 0.011, respectively) and decreased as the protein content increased (Figure 3). In sausages, the protein content had a stronger effect than for all other products combined (B = −0.070 and B = −0.111, respectively) (Table 3 and Table 4).

### 3.4. Evaluations of Risks and Benefits for Consumer Health

The food colours identified in fewer than five assortments (E100, E101 and E160A) and those unauthorised for usage in meat products and meat preparations (E153 and E171) were excluded from the analysis.

**Figure 4:**
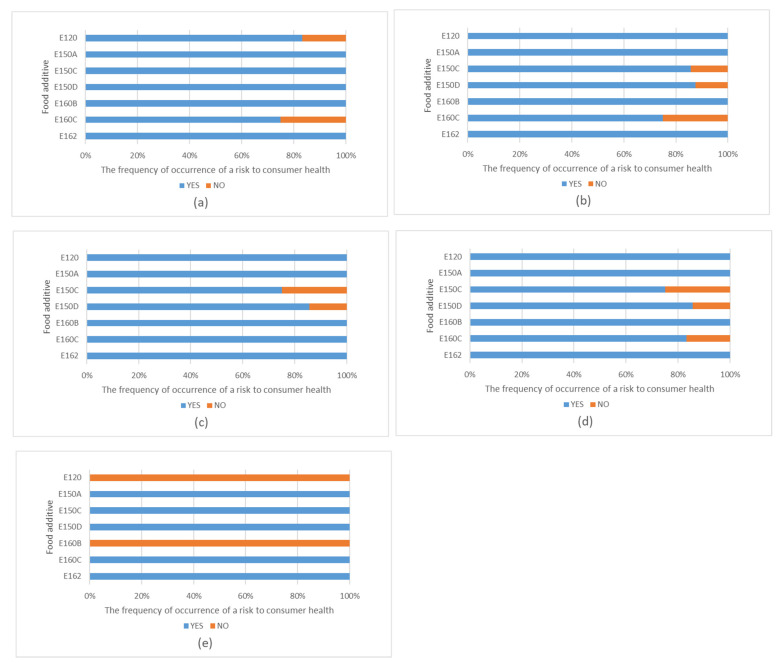
Evaluation of dyes in terms of the frequency of occurrence of a risk to consumer health. The health risks include (**a**) genotoxicity, (**b**) carcinogenicity, (**c**) acute toxicity, (**d**) chronic toxicity and (**e**) inducive potential for allergies.

Ninety-one reviewed publications were found to evaluate the risks and benefits for consumer health, i.e., genotoxicity, carcinogenicity, acute and chronic toxicity and an inducive potential towards allergies. The findings are illustrated in Figure 4.

## 4. Discussion

### 4.1. Carmine, Carminic Acid, Cochineal Extract—E120

Our findings indicate that the most commonly used dye in meat products and meat preparations is E120, also known as C.I. natural red 4. The main staining component of this natural red food additive is carminic acid. E120 is obtained by an aqueous, aqueous-alcohol or alcoholic extraction of the dried female specimens of *Dactylopius coccus Costa* (the cochineal beetle) [16,17,18].

The primary food categories contributing to exposure to E120 are as follows: soups and bouillons for infants, flavoured fermented milk products for infants and children, snacks and sauces for adolescents, and sauces, flavoured drinks, herbs and spices for adults and older adults. Importantly, although considerably less exposure is observed from category 8, i.e., meat and its preserves, this still represents a dozen or so per cent in respective age groups [18].

The FDA has classified Cochineal extract as a pigment exempt from certification, whose use is not liable to special restrictions [15]. In contrast, EFSA authorised this dye use in categories 8.2, 8.3.1 and 8.3.2 exclusively for specific assortment groups, with the preservation of maximum acceptable levels of use, except for Pasturma (air-dried cured beef) where it is allowed to stick to the *quantum satis* rule [7].

Our present findings indicate that E120 was used in accordance with regulations in the 164 sausage range and 2 pâté range. The additional visual inspection revealed that 7 out of the remaining 17 products did not contain an edible casing or decorations (8.3.3), suggesting that the use of the additive was incorrect [7].

Most previous studies show that both carminic acid and Cochineal extract are non-toxic, non-carcinogenic and non-genotoxic, and do not evoke either developmental or reproductive toxicity assuming an acceptable daily intake (ADI) of 5 mg/kg bm (carmine) and 2.5 mg/kg bm (carminic acid) [18,19,20,21]. Additionally, recent studies indicate that carminic acid might be an effective therapeutic agent used as part of the treatment against fructose-induced chronic renal damage [22].

However, a recent study by Arif, Ahmad and Ahmad indicates that carmine is a potentially cytotoxic, phytotoxic and genotoxic substance [23]. As such, its use with food may raise anxiety among consumers. Another paramount aspect for consumers is the fact that both carminic acid and carmine may trigger diverse allergic reactions in susceptible individuals, starting with mild pruritus [24], nettle rash, vasomotor oedema, atopic eczemas in children [25,26,27] and reaching acute hypersensitivity responses such as dyspnoea or bronchospasms that may lead to severe anaphylactic reactions [18,26,28,29,30,31,32,33]. Also, cases of hypersensitivity associated with long-term contact with the pigment were described. Examples cover rhinitis, conjunctivitis and asthma originating from professional exposure to carmine [29,34], e.g., the case of profession-related asthma in two butchers who used a mixture of spices with carmine for the production of sausages [35]. The most probable reason for manifesting this immunoglobulin E (IgE)-mediated allergic reaction is the contamination of the pigment with protein [32,34,36,37]. Our findings regarding the incidence of this dye in respective groups of products may help in making choices by consumers in whom the presence of E120-intake-induced allergic reactions occurred. Those consumers should largely avoid eating such products as luncheon meat and mett (raw-meat sausage), in which E120 is almost always present. In the case of lots such as fuet, salami and chorizo (varieties of sausages), the frequency of occurrence of carminic acid ranged from 40 to 54%.

### 4.2. Paprika Extract (E160c), Capsicum Extract, Capsanthin and Capsorubin

Our analysis of product labels found paprika extract (E160c) to be the second most commonly used food colouring in the studied meat products and preparations. It is a natural additive imparting a yellow to orange colour [3], also known as oleoresin from paprika. It is used in the form of a dark red viscous liquid, its major dyeing components being capsanthin and capsorubin. It is obtained by solvent-assisted extraction from the pods of numerous varieties of the *Capsicum annuum* (Linnaeus) pepper. The pigment may also contain capsaicin, i.e., chili extract, for which a limit of 250 mg/kg has been established [16]. The Joint FAO/WHO Expert Committee on Food Additives (JECFA) has fixed the ADI of paprika extract at the level of 1.5 mg/kg bw [38].

The EFSA exposure analysis indicates that the main food categories contributing to exposure to paprika extract used as a pigment are 6.3—Breakfast cereals, 7.2—Fine bakery wares and 12.5—Soups and broths. In contrast, exposure resulting from category 8—Meat and meat products, ranges from a few to over a dozen per cent in all age groups [39].

Paprika extract, E160c, is an additive belonging to group II-dyes approved for use in accordance with the principle of *quantum satis* (QS), except for the categories concerning meat preparations and meat products, where the maximum limit of use and products for which it can be used have been indicated [7]. However, the pigment is exempt from certification by the FDA, and its use is not liable to special restrictions [11]. In this study, thirty-one sausage products and six pâtés were found to have been treated with paprika extract as authorised. Regarding the other two assortments, visual assessment indicated that in one case, the paprika extract was used to decorate the meat (category 8.3.3), and was thus permitted, while the other had no decoration or edible casing, and hence was probably not [7].

Available studies indicate that paprika extract is non-toxic, non-carcinogenic, non-genotoxic [40,41,42] and probably does not evoke either developmental or reproductive toxicity assuming an intake below its ADI of 24 mg/kg bm [39]. In the case of capsaicin, older studies have indicated potential genotoxicity and carcinogenicity, while more recent studies based on purified capsaicin (not contaminated with other capsaicinoids) indicate low genotoxic and carcinogenic potential [43,44,45]; some even indicate a positive effect on human health. It is believed that this substance may have analgesic, antioxidant, anti-inflammatory and anticancer properties and could possibly be used to prevent obesity [46,47,48,49,50,51,52].

As there are no reports indicating that paprika extract has a negative impact on human health or any allergenic properties, and considering its antioxidant and colouring properties, this additive may be a natural substance that can replace or reduce the content of nitrites in meat products [53,54]. Studies show that approximately 3/4 of nitrites used in the production of sausages with regular fat content can be replaced with paprika oleoresin [54].

### 4.3. Betanin, Beetroot Red—E162

Betanin was found to be the third most commonly used dye in the tested meat preparations and meat products. It is obtained from the root of different beet varieties (*Beta vulgaris* L. var. rubra) by squeezing the juice from grated beets or by water extraction of shredded beetroot followed by enrichment with active ingredients [16,55,56]. In addition to its pigments, beetroot juice or extract contains sugars, salts and beet proteins [55].

The EFSA exposure analysis indicates that the main food categories contributing to exposure to betanin used as a pigment are 6.3—Breakfast cereals, 7.2—Fine bakery wares and 12.5—Soups and broths. In contrast, exposure resulting from category 8—Meat and meat products ranges from a few to over a dozen per cent in all age groups [55].

Beetroot Red is a group II additive, i.e., dyes approved for use based on the *quantum satis* principle, and is permitted for use in specific products in categories 8.2, 8.3.1 and 8.3.2 [7]. In contrast, the FDA regards beetroot red as a pigment exempt from certification, whose use is not liable to special restrictions [11]. The additive was observed in 29 of the tested assortments of which 26 were added in accordance with regulations: 25 sausages and 1 pâté. As the second product from the offals group was not a pâté, it was subjected to a visual assessment; the results indicate that it did not have an edible casing or meat decoration, and so the dye was probably misused. In turn, in the case of two products classified by the authors as meat preparations, unauthorised use could also be assumed. However, due to the lack of access to the producers’ records and thus the inability to clearly state that these sausages are meat preparations and not non-heat-treated processed meat for which such use of E162 would be correct, these assortments were not found to be non-compliant [7].

Beetroot red is non-toxic, non-genotoxic and non-carcinogenic [57,58,59,60]. However, the EFSA concluded that more comprehensive studies are needed to assess any potential genotoxicity, chronic toxicity and carcinogenicity and its impact on reproductive and developmental toxicity. In addition, no toxicological data are available to establish an acceptable daily intake for E162. However, exposure to betanin from the use of E162 as a food additive is considered to be in the same range as exposure to betanin from a regular diet; as such, betanin has been recognised as safe for use as a food additive [55]. Numerous studies indicate that betanin has a beneficial effect on human health. It acts as an antioxidant and anti-inflammatory agent, lowers blood pressure, restores the haemodynamics of brain vessels, supports the treatment of obesity, reveals cytotoxic properties concerning some cancer cells and is chemopreventive in relation to cancer [59,61,62,63,64,65,66,67]. In addition, the antioxidant properties of betanins effectively reduce oxidative stress in chronic heart failure, which indicates a cardioprotective effect [59,68]. In addition, beetroot red has been shown to have a cytotoxic effect on cancer cells, specifically human breast cancer cells: the use of the extract resulted in the activation of both intrinsic and extrinsic apoptosis pathways in breast cancer cells [59].

Due to their antioxidant activity, betanins may also be alternatives to synthetic antioxidants used to prevent lipid peroxidation processes in fat-containing foods, including meat products. Fat oxidation adversely affects the sensory properties, nutritional value and shelf life of products, and can also lead to the formation of compounds potentially harmful to health [59,69].

### 4.4. Caramel Colours 150a, 150c and 150d

The caramel dyes were found to rank fourth as a group among the tested food colourings used in processed meat. The group is further divided according to the reagents used in their production: Class I—plain caramel or caustic caramel (E150a), Class II—caustic sulphite caramel (E150b), Class III—ammonia caramel (E150c) and Class IV—ammonia sulphite caramel (E150d) [70,71].

Caramel or caustic caramel (E150a) is obtained by controlled heat treatment of carbohydrates, either monomers of glucose and fructose, or their polymers, such as glucose syrups, sucrose or inverted syrups and dextrose. This caramel is free of sulphite and ammonium compounds and produced by ordinary cooking processes. Therefore, there is no need to determine a numerical value of acceptable daily intake [71].

Ammonia caramel (E150c) is obtained by controlled heat treatment of carbohydrates, with or without adding acids or bases, in the presence of ammonium compounds (ammonium hydroxide, ammonium carbonate, ammonium bicarbonate and ammonium phosphate); sulphite compounds are not used [16,70]. The ADI set by the Scientific Committee on Food (SCF) was set at 200 mg/kg bw/day, assuming that the content of 2-acetyl-4-tetrahydroxybutylimidazole (THI) must not exceed 10 mg/kg of dye. Based on the intensity of the colour, the JECFA has established an ADI value of 25 mg THI/kg caramel colour [71].

Ammonia sulphite caramel (E150d) is obtained by controlled heat treatment of carbohydrates, with or without the addition of acids or bases, in the presence of both ammonium and sulphite compounds (sulphurous acid, potassium sulphite, potassium metabisulphite, sodium sulphite, sodium metabisulphite, ammonium hydroxide, ammonium bicarbonate, ammonium phosphate, ammonium sulphate, ammonium sulphite and ammonium bisulphite) [16]. For E150d, the SCF and the JECFA have set an ADI of 200 mg/kg bw/day.

Caramels are regarded as Group II additives, i.e., dyes approved for use in specific products within categories 8.2, 8.3.1 and 8.3 under the principle of *quantum satis* [7]. In contrast, the FDA regards caramel as being exempt from certification, whose use is not liable to special restrictions [11].

During the present study, it was found that caramels were used in the production of 38 assortments (1 sausage contained 2 types of caramel); among these, 22 were treated in accordance with the regulation: 14 were sausages and 8 were pâtés. The remaining 16 products, belonging to processed meat, were subjected to visual assessment on the basis of which it was established that in 8 cases, they had a casing or meat decoration (category 8.3.3), in which E150a-d is permitted. In the case of the other 8 products, no meat casing or decorations were found, so it is highly probable that the additive was misused [7].

Caramels I, III and IV do not appear to demonstrate acute or chronic toxicity, nor genotoxicity or carcinogenicity and do not appear to evoke any reproductive or developmental toxicity at the acceptable daily intake [72,73,74,75,76,77,78].

Doubts regarding the use of caramel III as a dye are caused by the immunotoxic effect of 2-acetyl-4-tetrahydroxybutylimidazole (THI), which is generated during its production. Human studies determined that THI did not affect the number of blood lymphocytes or the proliferation of the lymphocyte response to mitogenic stimulation and serum immunoglobulin levels. In the study, a type III caramel containing 23 ppm (commercial sample) or 143 ppm THI (study sample) was administered at an acceptable daily intake level of 200 mg/kg bw/day for seven days [79].

Concerns about the use of class III and IV caramel dyes also result from the identification of 4-methylimidazole in their composition, which is formed in the Maillard reaction as a result of the interaction of D-glucose and ammonia [80]. The National Toxicology Program (NTP) of the US National Institutes of Health, based on studies on the toxicity and carcinogenicity of 4-MEI and its structural isomer 2-MEI, found clear evidence that 4-MEI has carcinogenic activity in male and female B6C3F1 mice based on an increased incidence of alveolar or bronchiole cancer [76]. Accordingly, the International Agency for Research on Cancer has therefore concluded that 4-MI is “possibly carcinogenic to humans,” and The Office of Environmental Health Hazard Assessment (OEHHA) of the State of California’s Environmental Protection Agency has identified 4-MEI as a carcinogen [81].

In conclusion, the estimated consumption of caramel dyes depends largely on the eating habits of consumers. In the US, caramels are considered colourants exempt from certification and are used under general conditions without special restrictions; however, California regulations regard them as bearing potential carcinogenic effects [81]. In contrast, consumers in China receive relatively little exposure to class I, III and IV caramel dyes, with soy sauce, vinegar and spices contributing the most. Moreover, the risk of exposure to 4-MEI and THI from food colouring for the Chinese population was considered low based on current toxicology data [82].

### 4.5. Annatto, Bixin, Norbixin—E160b

The next most common colourant identified in the tested meat preparations and meat products was annatto (E160b). This substance is an orange-red natural dye that plays an important role as a pigment and additive in various industries and is obtained from the seeds of the tropical tree *Bixa orellana* [16,83]. The JECFA established an ADI for bixin of 0–12 mg/kg bw [84], and the SCF recently established an ADI of 6 mg bixin/kg bw per day and 0.3 mg norbixin/kg bw based on toxicological data [85].

A recent exposure analysis performed by the EFSA indicates that the main food category contributing to bixin-based annatto extract exposure among all age groups is 14.1.4—Flavoured drinks. Additionally, category 01.4—Flavoured fermented milk products are key sources of exposure among infants, children and adolescents, and 12.5—Soups and broths among the adult and elderly population. In contrast, the main categories contributing to exposure to norbixin-based annatto extract are the following: 07.2—Fine bakery wares for all age groups; category 06.3—Breakfast cereals and 12.5—Soups and broths for infants and children; categories 08.3—Meat products and 12.5—Soups and broths for adolescents and adults; and 06.3—Breakfast cereals, 08.3—Meat products and 12.5—Soups and broths for the elderly population [85,86].

E160B(i) bixin and E160B(ii) norbixin are authorised for use in certain types of products in categories 8.2, 8.3.1 and 8.3.2, subject to the maximum acceptable limit [7]. The FDA regards annatto extract as exempt from certification that can be used for colouring food in quantities consistent with good manufacturing practice [11]. During the present study, the additive was found in eight types of sausage; as it was approved for these categories, no incorrect use was found [7].

Most studies to date show that both bixin and norbixin are non-genotoxic, non-carcinogenic and non-toxic and do not evoke reproductive or developmental toxicity [87,88,89,90,91].

Our review of the literature indicated that annatto may cause allergic reactions. The possible symptoms include pruritus and hives [92,93,94], vasomotor oedema and even anaphylactic reactions [25,95,96,97]. This dye may also affect the severity of the symptoms of the disease in people with irritable bowel syndrome [98].

In addition, some studies indicate that the dye may have positive effects. Annatto supplementation in the diet has been proven to increase the resistance of human erythrocytes to haemolysis [99]. As a result of its antioxidant activity, this dye can also be considered a good source of natural antioxidants used in the production of meat products [100]. Additionally, adding annatto to processed meat products can partially replace the use of nitrites in production [101].

### 4.6. Assessment of the Frequency and Correctness of the Use of Dyes in Processed Meat Products

Dye was observed to be more than six times more common in sausages and more than three times in other meat products compared to smoked meats, offal products and meat preparations (Table 1); however, this is expected due to the restrictions indicated in Regulation 1333/2008 [7], governing the use of these dyes. All described dyes are permitted in food category no. 8, but their use was mainly limited to sausages (classified in categories 8.2, 8.3). These dyes could also be used in category 8.3.2 to produce terrines and pates, and additionally, annatto could also be used in luncheon meats; however, the dyes are not approved for use in smoked meat. Terrines and luncheon meats were qualified as *other meat products*. Therefore, it was predicted that dyes would be most commonly observed in sausages and that they would be absent in smoked meats; however, some dyes can be used in decorative casings and coatings, which will also apply to smoked meats.

Our analysis of the labels of processed meat products indicate the unauthorised use of dyes in 20 (7.33%) out of the 273 products in which they were used. Therefore, it seems reasonable to conduct further research to assess whether the dyes used in processed meat products are used at appropriate levels of use. Most discrepancies concerned the smoked meats group. In the authors’ opinion, smoked meats should be the first group that will be subjected to detailed chemical analysis.

It should be noted that any food on the market must be safe for human health [6]. Therefore, it is crucial that the FBO complies with Regulation 1333/2008 (EC), specifying the use of additives in food products, thereby preventing food safety hazards and ensuring that their products are safe. This is only possible if producers use authorised additives and observe specified maximum levels and conditions of use.

A positive correlation was found between the presence of water in meat products and the presence of dyes. This seems to be related to the way processed meat products are produced, i.e., traditional or conventional production [102]. Employing high-performance water-binding additives in the products, such as phosphates, carrageenan or starch, may contribute to this [102,103,104]. Therefore, it appears that dyes are less common in traditionally manufactured products than in conventional, high-performance products.

In addition, a positive correlation was noted between the occurrence of a dye and the fat contents per 100 g of the product, which suggests that high-fat products were found to be more likely to contain dyes. Undoubtedly, the fat content in processed meat products affects their colour. Research showed that reduced-fat products were redder compared with fatter products [105,106], while a higher protein level that is observed in lean meat—and thereby a higher level of myoglobin pigment in the product—significantly raised redness values [107]. The high-fat content products were darker and displayed browner colour, and they stood out with an increase in the proportion of yellow colour [108]. Higher fat content, as well as, for example, the addition of barley fibre, which also causes a significant darkening and an increase in the proportion of yellow colour of meat products, may result in poorer consumer acceptance of them [109]. In the authors’ opinion, therefore, it can be cautiously concluded that the desire to mask the unfavourable colour of fatty meat products may be the reason for the more frequent presence of dyes. Moreover, it should be remembered that meat is an important dietary source of saturated fatty acids (SFAs), which are responsible for the texture, juiciness and palatability of meat products [110]. However, due to the risk of many lifestyle diseases, including cardiovascular diseases, cancer and diabetes, consumers should avoid consuming products with a high SFA content [111]. The compliance of consumers with dietary recommendations and thus reducing the consumption of processed meat products with a high SFA content may probably lead to reducing exposure to dyes from these assortments.

Moreover, a positive correlation was found between the presence of a dye and the carbohydrates contents per 100 g of the product. In products where some of the meat proteins have been replaced by binders and fillers, such as rusk, breadcrumbs, cereal, legumes and soy protein, an increase in the carbohydrate content was observed. Food colourings in processed meat products are mainly used to camouflage fillers such as carbohydrates [112]. Therefore, probably, the more content of carbohydrates in these products, the higher is the need for such camouflage. Conversely, if products have a high protein content, they probably also have a lower carbohydrate content, and there is no need to improve the colour of the meat product, because the meat colour comes mainly from haem and myoglobin content [113]. Moreover, some colours are made from certain carbohydrates, e.g., caramel [114]; therefore, it is natural for the content of these ingredients and dyes to coincide.

### 4.7. Limitations

Our study has some limitations. First, due to our desire to obtain a comprehensive overview of the Polish market, and hence include as many samples as possible, our analyses were based on data obtained from processed meat product labels (the manufacturer’s declarations about used additives) rather than chemical analysis of the processed meat product. The processed meat products were not subjected to any analysis regarding the level of use of additives, because this information was not given on the labels. However, the degree of compliance of the products with legal requirements was determined based on the possibility of using to them dyes given on the labels. Therefore, as no chemical analysis was performed, and it was not possible to access the producers’ documents, the degree of compliance with the legal regulations may be overestimated.

## 5. Conclusions

Our findings indicate that 12 dyes are used in the production of meat products and preparations available on the Polish market. Out of the 273 studied assortments containing dyes, the most prolific pigment was found to be E120 carminic acid, found in 183 assortments. The presence of water and flavourings in the product increases the chances of the presence of a dye. As in the case where higher fat and carbohydrate contents per 100 g of the product increase the chance of a dye being present, this likelihood decreases as the protein content increases.

The results indicate a possible relationship between the presence of a dye in the product depending on the method of preparation (traditional or high-yielding products). In addition, dyes were more likely present in products with a higher carbohydrate content and less likely in products with higher protein content. The most significant number of inconsistencies was observed for smoked meats, and the greatest potential misuse of additives was noted for riboflavin and the caramel group. Most available analyses indicate no adverse effects on human health resulting from the use of food colourings, suggesting these additives are considered safe, but only if FBO complies with the regulations and guidelines on using food additives. Nevertheless, we cannot omit scientific reports that say that using E150C and E150D caramels can have potential carcinogenic effects and carmine and annatto can have allergic effects.

## Figures and Tables

**Table 1:** Frequency of dye presence in the studied meat preparations and meat products.

Assortment Group	Food Colouring (Number of Examples)											
	E100	E101	E120	E150A	E150C	E150D	E153	E160A	E160B	E160C	E162	E171
smoked meats	0	1	3	2	2	8	0	0	0	1	0	1
sausages	0	1	164	3	7	5	0	1	8	31	25	0
offal meats, including pâtés	2	2	2	6	1	5	0	0	0	6	2	0
other meat products	1	0	14	0	0	0	1	0	0	1	0	0
meat preparations	0	0	0	0	0	0	0	0	0	0	2	0
all products	3	4	183	11	10	18	1	1	8	39	29	1

**Table 2:** The percentage of non-compliance found in particular assortment groups.

Assortment Group	Food Colouring											
	E100	E101	E120	E150A	E150C	E150D	E153	E160A	E160B	E160C	E162	E171
smoked meats	0%	100%	33%	50%	50%	63%	0%	0%	0%	100%	0%	100%
sausages	0%	0%	0%	0%	0%	0%	0%	0%	0%	0%	0%	0%
offal meats, including pâtés	0%	0%	0%	0%	100%	0%	0%	0%	0%	0%	50%	0%
other meat products	0%	0%	43%	0%	0%	0%	100%	0%	0%	0%	0%	0%
meat preparations	0%	0%	0%	0%	0%	0%	0%	0%	0%	0%	0%	0%
all products	0%	25%	4%	9%	20%	28%	100%	0%	0%	3%	3%	100%

**Table 3:** Effect of water, flavours, product type, and fat, carbohydrate and protein content on the presence of dye in processed meat in a generalised linear binary model (*n* = 1834), 0*—reference category (B—beta coefficient, SE—standard error, Wald Chi^2^—chi square test of beta coefficient, *p*—*p* value of chi square test, Exp (B)—odds ratio, Lower CI—lower value of confidence interval, Upper CI—upper value of confidence interval).

Source	B	SE	Wald Chi^2^	*p*	Exp(B)	Lower CI	Upper CI
Intercept	−2.413	0.2748	77.117	0.000	0.090	0.052	0.153
PRODUCT (sausage)	1.901	0.2636	51.995	<0.001	6.690	3.991	11.215
PRODUCT (other meat products)	1.068	0.3787	7.955	0.005	2.910	1.385	6.112
PRODUCT (meat preparations)	0.702	0.7899	0.790	0.374	2.018	0.429	9.490
PRODUCT (offal meat)	0.409	0.3853	1.125	0.289	1.505	0.707	3.203
PRODUCT (smoked meats)	0*						
WATER (absent)	−0.591	0.1746	11.462	<0.001	0.554	0.393	0.780
WATER (present)	0*						
FAVOURS (absent)	−0.494	0.1555	10.082	0.001	0.610	0.450	0.828
FAVOURS (present)	0*						
FAT	0.031	0.0063	25.184	<0.001	1.032	1.019	1.045
CARBOHYDRATES	0.092	0.0282	10.568	0.001	1.096	1.037	1.158
PROTEIN	−0.070	0.0118	35.571	<0.001	0.932	0.911	0.954

**Table 4:** Effect of water and flavours, as well as fat, carbohydrate and protein content, on the presence of dye in sausages according to a generalised linear binary model (*n* = 937), 0*—reference category (B—beta coefficient, SE—standard error, Wald Chi^2^—chi square test of beta coefficient, *p*—*p* value of chi square test, Exp (B)—odds ratio, Lower CI—lower value of confidence interval, Upper CI—upper value of confidence interval).

Source	B	SE	Wald Chi^2^	*p*	Exp(B)	Lower CI	Upper CI
Intercept	−0.534	0.2026	6.959	0.008	0.586	0.394	0.872
WATER (absent)	−0.561	0.1967	8.119	0.004	0.571	0.388	0.839
WATER (present)	0*						
FAVOURS (absent)	−0.466	0.1825	6.510	0.011	0.628	0.439	0.898
FAVOURS (present)	0*						
FAT	0.058	0.0106	30.192	<0.001	1.060	1.038	1.082
CARBOHYDRES	0.093	0.0367	6.395	0.011	1.097	1.021	1.179
PROTEIN	−0.111	0.0182	37.128	<0.001	0.895	0.863	0.927

## Data Availability

The data presented in this study are available on request from the corresponding author.

## References

[1] Koch C., Koch E.C. (2003). Preconceptions of taste based on color. J. Psychol..

[2] Wadhera D., Capaldi-Phillips E.D. (2014). A review of visual cues associated with food on food acceptance and consumption. Eat. Behav..

[3] Silva M.M., Reboredo F.H., Lidon F.C. (2022). Food Colour Additives: A Synoptical Overview on Their Chemical Properties, Applications in Food Products, and Health Side Effects. Foods.

[4] Spence C., Wan X., Woods A., Velasco C., Deng J., Youssef J., Deroy O. (2015). On tasty colours and colourful tastes? Assessing, explaining and utilizing crossmodal correspondences between colours and basic tastes. Flavour.

[5] Spence C., Levitan C.A., Shankar M.U., Zampini M. (2010). Does Food Color Influence Taste and Flavor Perception in Humans?. Chemosens. Percept..

[6] EC (2002). Regulation (EC) No. 178/2002 of the European Parliament and of the Council of 28 January 2002. Laying down the general principles and requirements of food law, establishing the European Food Safety Authority and laying down procedures in matters of food safety. Off. J. Eur. Union.

[7] EC (2008). Regulation (EC) No. 1333/2008 of 16 December 2008. On Food Additives. Off. J. Eur. Union.

[8] Guidance (EC) Guidance Document Describing the Food Categories in Part E of Annex II to Regulation (EC) No 1333/2008 on Food Additives. https://food.ec.europa.eu.

[9] EC (2004). Regulation (EC) No. 853/2004 of the European Parliament and of the Council of 29 April 2004. Laying down specific hygiene rules for food of animal origin. Off. J. Eur. Union.

[10] CFR Color Additives. 21 CFR Part 70. https://www.ecfr.gov/current/title-21/chapter-I/subchapter-A/part-70.

[11] CFR Listing of Color Additives Exempt from Certification. 21 CFR Part 73. https://www.ecfr.gov/current/title-21/chapter-I/subchapter-A/part-73.

[12] PKN (1996). Polska Norma PN-A-82007.

[13] MF Dane z Zeznań Podatkowych Podatników, o Których Mowa w Art. 27b Ustawy z Dnia 15 Lutego 1992 r. o Podatku Dochodowym od Osób Prawnych (Dz. U. z 2017 r. poz. 2343, ze zm.) Oraz w Ustawie z 24 Listopada 2017 r. o Zmianie Ustawy o Podatku Dochodowym od Osób Prawnych (Dz. U. poz. 2369). https://gov.pl.

[14] GUS Demographic Yearbook of Poland 2018. https://stat.gov.pl.

[15] Dobson A.J., Adrian G.B. (2018). An Introduction to Generalized Linear Models.

[16] EU (2012). Commission Regulation (EU) No. 231/2012 of 22 March 2012. Laying Down Specifications for Food Additives Listed in Annexes II and III to Regulation (EC) No. 1333/2008 of the European Parliament and of the Council. J. Eur. Union.

[17] FAO Cochineal Extract. Monograph 1. Prepared at the 55th JECFA 2000. https://www.fao.org/fileadmin/user_upload/jecfa_additives/docs/Monograph1/Additive-137.pdf.

[18] EFSA Panel on Food Additives and Nutrient Sources added to Food (ANS) (2015). Scientific Opinion on the re-evaluation of cochineal, carminic acid, carmines (E120) as a food additive. EFSA J..

[19] Cooksey C.J. (2019). The red insect dyes: Carminic, kermesic and laccaic acids and their derivatives. Biotech. Histochem..

[20] Merinas-Amo R., Martínez-Jurado M., Jurado-Güeto S., Alonso-Moraga Á., Merinas-Amo T. (2019). Biological Effects of Food Coloring in In Vivo and In Vitro Model Systems. Foods.

[21] Sarıkaya R., Selvi M., Erkoç F. (2012). Evaluation of potential genotoxicity of five food dyes using the somatic mutation and recombination test. Chemosphere.

[22] Li Q., Xu Q., Tan J., Hu L., Ge C., Xu M. (2021). Carminic acid supplementation protects against fructose-induced kidney injury mainly through suppressing inflammation and oxidative stress via improving Nrf-2 signaling. Aging.

[23] Arif A., Ahmad A., Ahmad M. (2021). Toxicity assessment of carmine and its interaction with calf thymus DNA. J. Biomol. Struct. Dyn..

[24] Lucas C.D., Hallagan J.B., Taylor S.L. (2001). The role of natural color additives in food allergy. Adv. Food Nutr. Res..

[25] Andreozzi L., Giannetti A., Cipriani F., Caffarelli C., Mastrorilli C., Ricci G. (2019). Hypersensitivity reactions to food and drug additives: Problem or myth?. Acta Biomed..

[26] Chung K., Baker J.R., Baldwin J.L., Chou A. (2001). Identification of carmine allergens among three carmine allergy patients. Allergy.

[27] Lemoine A., Pauliat-Desbordes S., Challier P., Tounian P. (2020). Adverse reactions to food additives in children: A retrospective study and a prospective survey. Arch. Pediatr..

[28] Beaudouin E., Kanny G., Lambert H., Fremont S., Moneret-Vautrin D.A. (1995). Food anaphylaxis following ingestion of carmine. Ann. Allergy Asthma Immunol..

[29] Greenhawt M.J., Baldwin J.L. (2009). Carmine dye and cochineal extract: Hidden allergens no more. Ann. Allergy Asthma Immunol..

[30] Kägi M.K., Wüthrich B., Johansson S.G. (1994). Campari-Orange anaphylaxis due to carmine allergy. Lancet.

[31] Takeo N., Nakamura M., Nakayama S., Okamoto O., Sugimoto N., Sugiura S., Sato N., Harada S., Yamaguchi M., Mitsui N. (2018). Cochineal dye-induced immediate allergy: Review of Japanese cases and proposed new diagnostic chart. Allergol. Int..

[32] Wüthrich B., Kägi M.K., Stücker W. (1997). Anaphylactic reactions to ingested carmine (E120). Allergy.

[33] Yamakawa Y., Oosuna H., Yamakawa T., Aihara M., Ikezawa Z. (2009). Cochineal extract-induced immediate allergy. J. Dermatol..

[34] Gultekin F., Doguc D.K. (2013). Allergic and immunologic reactions to food additives. Clin. Rev. Allergy Immunol..

[35] Añíbarro B., Seoane J., Vila C., Múgica V., Lombardero M. (2003). Occupational asthma induced by inhaled carmine among butchers. Int. J. Occup. Med. Environ. Health.

[36] DiCello M.C., Myc A., Baker J.R., Baldwin J.L. (1999). Anaphylaxis after ingestion of carmine colored foods: Two case reports and a review of the literature. Allergy Asthma Proc..

[37] Ohgiya Y., Arakawa F., Akiyama H., Yoshioka Y., Hayashi Y., Sakai S., Ito S., Yamakawa Y., Ohgiya S., Ikezawa Z. (2009). Molecular cloning, expression, and characterization of a major 38-kd cochineal allergen. J. Allergy Clin. Immunol..

[38] FAO Paprika Extract. Monograph 14. Prepared at the 77th JECFA 2013. https://www.fao.org/fileadmin/user_upload/jecfa_additives/docs/monograph16/additive-510-m16.pdf.

[39] Scientific Opinion (2017). On the re-evaluation of paprika extract (E160c) as a food additive. In: EFSA Panel on Food Additives and Nutrient Sources added to Food (ANS). EFSA J..

[40] Akagi A., Sano N., Uehara H., Minami T., Otsuka H., Izumi K. (1998). Non-carcinogenicity of capsaicinoids in B6C3F1 mice. Food Chem. Toxicol..

[41] Inoue T., Umemura T., Maeda M., Ishii Y., Okamura T., Tasaki M., Nishikawa A. (2008). Safety assessment of dietary administered paprika color in combined chronic toxicity and carcinogenicity studies using F344 rats. Food Chem. Toxicol..

[42] Kanki K., Nishikawa A., Furukawa F., Kitamura Y., Imazawa T., Umemura T., Hirose M. (2003). A 13-week subchronic toxicity study of paprika color in F344 rats. Food Chem. Toxicol..

[43] Bley K., Boorman G., Mohammad B., McKenzie D., Babbar S. (2012). A comprehensive review of the carcinogenic and anticarcinogenic potential of capsaicin. Toxicol. Pathol..

[44] Chanda S., Erexson G., Riach C., Innes D., Stevenson F., Murli H., Bley K. (2004). Genotoxicity studies with pure trans-capsaicin. Mutat. Res..

[45] Díaz Barriga Arceo S., Madrigal-Bujaidar E., Calderón Montellano E., Ramírez Herrera L., Díaz García B.D. (1995). Genotoxic effects produced by capsaicin in mouse during subchronic treatment. Mutat. Res..

[46] Baskaran P., Krishnan V., Ren J., Thyagarajan B. (2016). Capsaicin induces browning of white adipose tissue and counters obesity by activating TRPV1 channel-dependent mechanisms. Br. J. Pharmacol..

[47] Baskaran P., Markert L., Bennis J., Zimmerman L., Fox J., Thyagarajan B. (2019). Assessment of Pharmacology, Safety, and Metabolic activity of Capsaicin Feeding in Mice. Sci. Rep..

[48] Chapa-Oliver A.M., Mejía-Teniente L. (2016). Capsaicin: From Plants to a Cancer-Suppressing Agent. Molecules.

[49] Cho S.C., Lee H., Choi B.Y. (2017). An updated review on molecular mechanisms underlying the anticancer effects of capsaicin. Food Sci. Biotechnol..

[50] Chung Y.C., Baek J.Y., Kim S.R., Ko H.W., Bok E., Shin W.H., Won S.Y., Jin B.K. (2017). Capsaicin prevents degeneration of dopamine neurons by inhibiting glial activation and oxidative stress in the MPTP model of Parkinson’s disease. Exp. Mol. Med..

[51] Höper J., Helfert S., Heskamp M.L., Maihöfner C.G., Baron R. (2014). High concentration capsaicin for treatment of peripheral neuropathic pain: Effect on somatosensory symptoms and identification of treatment responders. Curr. Med. Res. Opin..

[52] Kim C.S., Kawada T., Kim B.S., Han I.S., Choe S.Y., Kurata T., Yu R. (2003). Capsaicin exhibits anti-inflammatory property by inhibiting IkB-a degradation in LPS-stimulated peritoneal macrophages. Cell. Signal..

[53] Bázan-Lugo E., García-Martínez I., Alfaro-Rodríguez R.H., Totosaus A. (2012). Color compensation in nitrite-reduced meat batters incorporating paprika or tomato paste. J. Sci. Food Agric..

[54] Kim G.H., Chin K.B. (2021). Characteristics of low-nitrite pork emulsified-sausages with paprika oleoresin solution during refrigerated storage. J. Anim. Sci. Technol..

[55] Scientific Opinion (2015). On the re-evaluation of beetroot red (E 162) as a food additive. In: EFSA Panel on Food Additives and Nutrient Sources added to Food (ANS). EFSA J..

[56] FAO Beet Red. Monograph. Prepared at the 31st JECFA 1987. https://www.fao.org/fileadmin/user_upload/jecfa_additives/docs/Monograph1/Additive-052.pdf.

[57] Haveland-Smith R.B. (1981). Evaluation of the genotoxicity of some natural food colours using bacterial assays. Mutat. Res..

[58] Reynoso R.C., Giner T.V., de Mejia E.G. (1999). Safety of a filtrate of fermented garambullo fruit: Biotransformation and toxicity studies. Food Chem. Toxicol..

[59] Sadowska-Bartosz I., Bartosz G. (2021). Biological Properties and Applications of Betalains. Molecules.

[60] von Elbe J.H., Schwartz S.J. (1981). Absence of mutagenic activity and a short-term toxicity study of beet pigments as food colorants. Arch. Toxicol..

[61] Clifford T., Howatson G., West D.J., Stevenson E.J. (2015). The potential benefits of red beetroot supplementation in health and disease. Nutrients.

[62] Esatbeyoglu T., Wagner A.E., Schini-Kerth V.B., Rimbach G. (2015). Betanin—A food colorant with biological activity. Mol. Nutr. Food Res..

[63] Georgiev V.G., Weber J., Kneschke E.M., Denev P.N., Bley T., Pavlov A.I. (2010). Antioxidant activity and phenolic content of betalain extracts from intact plants and hairy root cultures of the red beetroot Beta vulgaris cv. Detroit dark red. Plant Foods Hum. Nutr..

[64] Gliszczyńska-Swigło A., Szymusiak H., Malinowska P. (2006). Betanin, the main pigment of red beet: Molecular origin of its exceptionally high free radical-scavenging activity. Food Addit. Contam..

[65] Khan M.I. (2016). Plant Betalains: Safety, Antioxidant Activity, Clinical Efficacy, and Bioavailability. Compr. Rev. Food Sci. Food Saf..

[66] Lechner J.F., Stoner G.D. (2019). Red Beetroot and Betalains as Cancer Chemopreventative Agents. Molecules.

[67] Zielińska-Przyjemska M., Olejnik A., Dobrowolska-Zachwieja A., Grajek W. (2009). In vitro effects of beetroot juice and chips on oxidative metabolism and apoptosis in neutrophils from obese individuals. Phytother. Res..

[68] Gao Y., Liang X., Tian Z., Ma Y., Sun C. (2021). Betalain exerts cardioprotective and anti-inflammatory effects against the experimental model of heart failure. Hum. Exp. Toxicol..

[69] Vieira Teixeira da Silva D., Dos Santos Baião D., de Oliveira Silva F., Alves G., Perrone D., Mere Del Aguila E., Paschoalin V.M.F. (2019). Betanin, a Natural Food Additive: Stability, Bioavailability, Antioxidant and Preservative Ability Assessments. Molecules.

[70] FAO Caramel Colours. Monograph 11. Prepared at the 74th JECFA 2011. https://www.fao.org/fileadmin/user_upload/jecfa_additives/docs/monograph11/additive-329-m11.pdf.

[71] Scientific Opinion (2011). On the re-evaluation of caramel colours (E 150 a,b,c,d) as food additives. In: EFSA Panel on Food Additives and Nutrient Sources added to Food (ANS). EFSA J..

[72] Adams K., Allen J.A., Brooker P.C., Jones E., Proudlock R.J. (1992). Assessment of the genotoxic potential of Caramel Colour I in four short-term tests. Food Chem. Toxicol..

[73] Allen J.A., Brooker P.C., Jones E., Adams K., Richold M. (1992). Absence of mutagenic activity in Salmonella and of clastogenic activity in CHO cells of Caramel Colours I, II, III and IV. Food Chem. Toxicol..

[74] Brusick D.J., Jagannath D.R., Galloway S.M., Nestmann E.R. (1992). Genotoxicity hazard assessment of Caramel Colours III and IV. Food Chem. Toxicol..

[75] Houben G.F., Penninks A.H. (1994). Immunotoxicity of the colour additive caramel colour III; a review on complicated issues in the safety evaluation of a food additive. Toxicology.

[76] National Toxicology Program (2007). Toxicology and carcinogenesis studies of 4-methylimidazole (Cas No. 822-36-6) in F344/N rats and B6C3F1 mice (feed studies). Natl. Toxicol. Program Tech. Rep. Ser..

[77] Sengar G., Sharma H.K. (2014). Food caramels: A review. J. Food Sci. Technol..

[78] Vollmuth T.A. (2018). Caramel color safety—An update. Food Chem. Toxicol..

[79] Houben G.F., Abma P.M., van den Berg H., van Dokkum W., van Loveren H., Penninks A.H., Seinen W., Spanhaak S., Vos J.G., Ockhuizen T. (1992). Effects of the colour additive caramel colour III on the immune system: A study with human volunteers. Food Chem. Toxicol..

[80] Hengel M., Shibamoto T. (2013). Carcinogenic 4(5)-methylimidazole found in beverages, sauces, and caramel colors: Chemical properties, analysis, and biological activities. J. Agric. Food Chem..

[81] Jacobson M.F. (2012). Carcinogenicity and regulation of caramel colorings. Int. J. Occup. Med. Environ. Health.

[82] Liang J., Cao P., Wang X., Gao P., Xu H., Ma N. (2019). Dietary intake assessment of caramel colours and their processing by-products 4-methylimidazole and 2-acetyl-4-tetrahydroxy-butylimidazole for the Chinese population. Food Addit. Contam..

[83] Giuliano G., Rosati C., Bramley P.M. (2003). To dye or not to dye: Biochemistry of annatto unveiled. Trends Biotechnol..

[84] FAO Annatto Extract (Solvent-Extracted Bixin). Monograph 17. Prepared at the 80th JECFA 2015. https://www.fao.org/fileadmin/user_upload/jecfa_additives/docs/monograph17/additive-040-m17.pdf.

[85] Younes M., Castle L., Engel K.H., Fowler P., Frutos Fernandez M.J., Fürst P., Gürtler R., Gundert-Remy U., Husøy T., Mennes W. (2019). Safety of annatto E and the exposure to the annatto colouring principles bixin and norbixin (E 160b) when used as a food additive. In: EFSA Panel on Food Additives and Flavourings (FAF). EFSA J..

[86] Tard A., EFSA (2017). Exposure assessment of annatto colouring principles bixin and norbixin (E 160b) when used as food additives. EFSA J..

[87] Agner A.R., Barbisan L.F., Scolastici C., Salvadori D.M. (2004). Absence of carcinogenic and anticarcinogenic effects of annatto in the rat liver medium-term assay. Food Chem. Toxicol..

[88] Bautista A.R., Moreira E.L., Batista M.S., Miranda M.S., Gomes I.C. (2004). Subacute toxicity assessment of annatto in rat. Food Chem. Toxicol..

[89] Júnior A.C., Asad L.M., Oliveira E.B., Kovary K., Asad N.R., Felzenszwalb I. (2005). Antigenotoxic and antimutagenic potential of an annatto pigment (norbixin) against oxidative stress. Genet. Mol. Res..

[90] Paumgartten F.J., De-Carvalho R.R., Araujo I.B., Pinto F.M., Borges O.O., Souza C.A., Kuriyama S.N. (2002). Evaluation of the developmental toxicity of annatto in the rat. Food Chem. Toxicol..

[91] Scientific Opinion (2016). On the safety of annatto extracts (E 160b) as a food additive. In: EFSA Panel on Food Additives and Nutrient Sources added to Food (ANS). EFSA J..

[92] Myles I.A., Beakes D. (2009). An Allergy to Goldfish? Highlighting the Labeling Laws for Food Additives. World Allergy Organ. J..

[93] Ramsey N.B., Tuano K.T., Davis C.M., Dillard K., Hanson C. (2016). Annatto seed hypersensitivity in a pediatric patient. Ann. Allergy Asthma Immunol..

[94] Sadowska B., Sztormowska M., Chełmińska M. (2021). Annatto hypersensitivity after oral ingestion confirmed by placebo-controlled oral challenge. Ann. Allergy Asthma Immunol..

[95] Ebo D.G., Ingelbrecht S., Bridts C.H., Stevens W.J. (2009). Allergy for cheese: Evidence for an IgE-mediated reaction from the natural dye annatto. Allergy.

[96] Nish W.A., Whisman B.A., Goetz D.W., Ramirez D.A. (1991). Anaphylaxis to annatto dye: A case report. Ann. Allergy.

[97] Randhawa S., Bahna S.L. (2009). Hypersensitivity reactions to food additives. Curr. Opin. Allergy Clin. Immunol..

[98] Stein H.L. (2009). Annatto and IBS. J. Clin. Gastroenterol..

[99] Beni A.A., Rodrigues R.F., Conte L., Costa I.F., Delalibera É.A., Roehrs M., Rampelotto C., Emanuelli T., Somacal S. (2020). Dietary supplementation with annatto food-coloring extracts increases the resistance of human erythrocytes to hemolysis. Nutr. Res..

[100] Cuong T.V., Chin K.B. (2016). Effects of Annatto (*Bixa orellana* L.) Seeds Powder on Physicochemical Properties, Antioxidant and Antimicrobial Activities of Pork Patties during Refrigerated Storage. Korean J. Food Sci. Anim. Resour..

[101] Zarringhalami S., Sahari M.A., Hamidi-Esfehani Z. (2009). Partial replacement of nitrite by annatto as a colour additive in sausage. Meat Sci..

[102] Halagarda M., Wójciak K.M. (2022). Health and safety aspects of traditional European meat products. A review. Meat Sci..

[103] Halagarda M., Kędzior W., Pyrzyńska E. (2017). Nutritional Value and Potential Chemical Food Safety Hazards of Selected Traditional and Conventional Pork Hams from Poland. J. Food Qual..

[104] Halagarda M., Kędzior W., Pyrzyńska E. (2018). Nutritional value and potential chemical food safety hazards of selected Polish sausages as influenced by their traditionality. Meat Sci..

[105] Crehan C.M., Hughes E., Troy D.J., Buckley D.J. (2000). Effects of fat level and maltodextrin on the functional properties of frankfurters formulated with 5, 12 and 30% fat. Meat Sci..

[106] Hughes E., Cofrades S., Troy D.J. (1997). Effects of fat level, oat fibre and carrageenan on frankfurters formulated with 5, 12 and 30% fat. Meat Sci..

[107] Youssef M.K., Barbut S. (2009). Effects of protein level and fat/oil on emulsion stability, texture, microstructure and color of meat batters. Meat Sci..

[108] Sarıçoban C., Yılmaz M.T. (2010). Modelling the Effects of Processing Factors on the Changes in Colour Parameters of Cooked Meatballs Using Response Surface Methodology. World Appl. Sci. J..

[109] Słowiński M., Miazek J., Dasiewicz K., Chmiel M. (2021). The Effect of the Addition of Fiber Preparations on the Color of Medium-Grounded Pasteurized and Sterilized Model Canned Meat Products. Molecules.

[110] Barbut S., Wood J., Marangoni A. (2016). Potential use of organogels to replace animal fat in comminuted meat products. Meat Sci..

[111] Islam M.A., Amin M.N., Siddiqui S.A., Hossain M.P., Sultana F., Kabir M.R. (2019). Trans fatty acids and lipid profile: A serious risk factor to cardiovascular disease, cancer and diabetes. Diabetes Metab. Syndr..

[112] Babji A.S., Nuri M.N., Suherman J., Seri Chempaka M.Y. (2000). Quality assessment of local and franchise beef and chicken burgers. Pertanika J. Trop. Agric..

[113] Downham A., Collins P. (2000). Colouring our foods in the last and next millennium. Int. J. Food Sci..

[114] Wideman N., O’bryan C.A., Crandall P.G. (2016). Factors affecting poultry meat colour and consumer preferences-A review. Worlds Poult. Sci. J..

